# Protocol for lipid mediator profiling and phenotyping of human M1- and M2-monocyte-derived macrophages during host-pathogen interactions

**DOI:** 10.1016/j.xpro.2024.103142

**Published:** 2024-09-17

**Authors:** Kerstin Günther, Christina Ehrhardt, Oliver Werz, Paul M. Jordan

**Affiliations:** 1 Department of Pharmaceutical/Medicinal Chemistry, Institute of Pharmacy, Friedrich Schiller University Jena, Philosophenweg 14, 07743 Jena, Germany; 2 Section of Experimental Virology, Institute of Medical Microbiology, Center for Molecular Biomedicine (CMB), Jena University Hospital, Hans-Knoell-Str. 2, 07745 Jena, Germany; 3 Jena Center for Soft Matter (JCSM), Friedrich Schiller University Jena, Philosophenweg 7, 07743 Jena, Germany

**Keywords:** Cell Biology, Cell isolation, Cell-based Assays, Immunology, Microbiology

## Abstract

Here, we present a protocol for primary, human immune cell isolation and stimulation for lipid mediator profiling. We describe steps for the isolation of monocytes from human leukocyte concentrates via density centrifugation and differentiation/polarization toward M1- or M2-monocyte-derived macrophages (MDMs). We detail stimulation approaches of MDMs with live bacteria or influenza A virus for lipid mediator profiling and sample preparation for subsequent analysis, such as enzyme expression, mRNA analysis, or surface marker determination.

For complete details on the use and execution of this protocol, please refer to Jordan et al.^1^

## Highlights

• Steps for isolating peripheral blood mononuclear cells from leukocyte concentrates
• Instructions for differentiation and polarization of monocyte-derived macrophages
• Classification of different seeding procedures for short-term and long-term incubations
• Stimulation procedure for lipid mediator profiling of human M1- and M2-MDMs

## Abstract (editor highlights)

Publisher’s note: Undertaking any experimental protocol requires adherence to local institutional guidelines for laboratory safety and ethics.

## Graphical abstract

**Figure:**
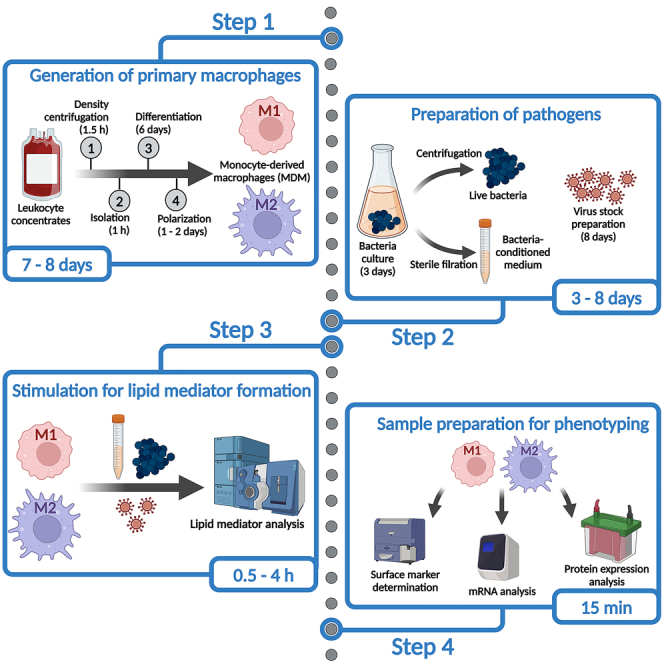

## Before you begin

The presented protocol has been developed for broad analysis of human monocyte-derived macrophage (MDM) phenotyping and lipid mediator (LM) profiling after host-pathogen interactions. To generate human MDMs, leukocyte concentrates derived from freshly withdrawn blood (16 I.E. heparin/mL blood) of healthy adult male and female volunteers (18–65 years, without details about ancestry, race or ethnicity) were provided, according to guidelines and regulations for working with human material. All steps were performed in a cell culture lab under sterile conditions. This protocol is standardized for high yield isolation of MDMs from different blood donations of the same day. For cell isolation, we prefer using electronic pipettors for time-saving handling. The used solutions and consumables, if not stated otherwise, were all sterile or sterilized via autoclaving. For preparation of all pathogens and pathogen components authorization is needed to handle the pathogens according to the guidelines and laws in your country.

### Institutional permissions

The present protocol uses human leukocyte concentrates obtained from freshly withdrawn peripheral blood of healthy adult human donors. The institutions have to be authorized for working with human material and also for handling of human pathogenic organisms. Note that replicating the present protocol with human samples requires the permission of the Ethical Review Body of the relevant institution.

## Key resources table

**Table:** 

REAGENT or RESOURCE	SOURCE	IDENTIFIER
**Bacterial and virus strains**		

*S. aureus*/USA300	Kindly provided by Dr. Lorena Tuchscherr, Jena University Hospital, Germany	N/A
*S. aureus*/6850	Kindly provided by Dr. Lorena Tuchscherr, Jena University Hospital, Germany	N/A
*E. coli/*O6:K2:H1	ATCC	Cat#19138
Influenza A virus/Puerto Rico/8/34 (H1N1, PR8)	Kindly provided by Dr. Stephan Ludwig, Institute of Virology, University of Muenster, Germany	N/A

**Chemicals, peptides, and recombinant proteins**		

Acetic acid	Sigma	Cat#A6283; CAS: 64-19-7
Albumin solution 30% (BA)	Roth	Cat#9401
Brain-heart-infusion broth	Sigma	Cat#53286
Bovine serum albumin (BSA)	Roth	Cat#T844.3
Columbia-Agar with sheep blood (5%) plate	Thermo Scientific	Cat#10463833
CaCl_2_	AppliChem	Cat#A1428; CAS: 10043-52-4
d4-LTB_4_	Cayman Chemical	Cat#Cay320110; CAS: 124629-74-9
d4-PGE_2_	Cayman Chemical	Cat#Cay314010; CAS: 34210-10-1
d5-LXA_4_	Cayman Chemical	Cat#Cay10007737; CAS: 1622429-53-1
d5-RvD2	Cayman Chemical	Cat#Cay11184; CAS: 1881277-33-3
d8-5S-HETE	Cayman Chemical	Cat#Cay334230; CAS: 330796-62-8
d8-AA	Cayman Chemical	Cat#Cay390010; CAS: 69254-37-1
DMSO	VWR	Cat#1029500500; CAS: 67-68-5
DEAE-Dextran	Sigma	Cat#D9885; CAS: 9064-91-9
Dextran from *Leuconostoc* spp.	Sigma	Cat#31392; CAS: 9004-54-0
Dulbecco’s phosphate-buffered saline with calcium chloride and magnesium chloride 10×	Sigma	Cat#D1283
Eagle’s minimum essential medium (EMEM)	Sigma	Cat#M4526
Fetal calf serum (FCS)	Sigma	Cat#F7524
β-Glycerophosphate disodium salt hydrate	Sigma	Cat#5422; CAS: 13408-09-8
GM-CSF	PeproTech	Cat#300-23; GenPept: P04141
L-glutamine	Sigma	Cat#G7513; CAS: 56-85-9
Histopaque-1077	Sigma	Cat#10771; CAS: 97639-11-7
IFN-γ	PeproTech	Cat#300-02; GenPept: P01579.1
IL-4	PeproTech	Cat#200-04; GenPept: P05112
Leukocyte pack	Jena University Hospital, Germany	N/A
Lipopolysaccharide (*E. coli*)	Sigma	Cat#L3129
Lymphoprep	Fisher Scientific	Cat#NC0665098
M-CSF	PeproTech	Cat#300-25; GenPept: P09603
Methanol	Thermo Fisher Scientific	Cat#325740010; CAS: 67-56-1
β-Mercaptoethanol	Sigma	Cat#M3148; CAS: 60-24-2
MgCl_2_	VWR	Cat#1,05833; CAS: 7786-30-3
Na_4_P_2_O_7_ × 10H_2_O	Sigma	Cat#PHR2205; CAS:13472-36-1
Na_3_VO_4_	Sigma	Cat#450243; CAS: 13721-39-6
NaCl	Roth	Cat#HN00.3; CAS: 7647-14-5
NaF	AppliChem	Cat#A3904,0025; CAS: 7681-49-4
Neutral Red	Sigma	Cat#N4638; Cas: 553-24-2
Nonidet P40 (NP-40)	AppliChem	Cat#A1694,0250; CAS: 9002-93-1
Nutrient broth	Sigma	Cat#70122
Oxoid purified agar	Thermo Fisher Scientific	Cat#LP0028B
Panserin 401	PAN-Biotech	Cat#P04-710401
Phosphate-buffered saline	Serva	Cat#47302.03
Penicillin/streptomycin	Sigma	Cat#P0781
RPMI 1640	Sigma	Cat#R8758
Sodium azide	Sigma	Cat#8.22335.0250; CAS: 26628-22-8
Sodium bicarbonate (NaHCO_3_) 7.5% solution	Thermo Fisher Scientific	Cat#25080102
TPCK-trypsin	Sigma	Cat#T1426
TRIS	Roth	Cat#AE15.3

**Experimental models: Cell lines**		

MDCK II cells	Kindly provided by Dr. Stephan Ludwig, Institute of Virology, University of Muenster, Germany	N/A
	ATCC	Cat#CRL-2936

**Critical commercial assays**		

DC Protein Assay Reagents Package	Bio-Rad	Cat#5000116
E.Z.N.A. Total RNA Kit	Omega BioTek	Cat#R6834-02

**Other**		

15 mL Falcon	Greiner	Cat#188271
50 mL Falcon	Greiner	Cat#227261
175 cm^2^ cell culture flask	Greiner	Cat#661160
Cell scraper	Greiner	Cat#541070
6-well plate	Greiner	Cat#657160
12-well plate	Greiner	Cat#665180
24-well pate	Greiner	Cat#662160
96-well plate	Greiner	Cat#10412741
15 cm plate	Sigma	Cat#CLS430599
DURAN Erlenmeyer flask, NS 29/32, 100 mL	DWK Life Sciences	Cat#241932705
Reaction tube, 1.5 mL (RNA-free)	Greiner	Cat#616201
Optical density tube	Sarstedt AG & Co. KG	Cat#62.515.006
Hard loop 1 μL sterile	VWR	Ca#612-9352
Rotilabo - syringe filters, PVDF, sterile, 0.22 μm	Roth	Cat#P666.1
Disposable syringe Omnifix with Luer-lock fitting, 10 mL	Roth	Cat#C542.1
Disposable needles Sterican long bevel facet, 70 mm, 0.90 mm, yellow	Roth	Cat#C722.1
Heraeus Multifuge X3R centrifuge	Thermo Fisher Scientific	
Vi-Cell XR cell counter	Beckman Coulter	
Ultrospec 10 cell density meter	Amersham Biosciences	
BioPhotometer plus	Eppendorf	
Heraguard laminar air flow bench	Thermo Fisher Scientific	
Model CB160 incubator	Binder	
Reinstwassersystem TKA GenPure inkl. xCAD-Dispenser	Huber Lab	
Zeiss Axiovert 25 inverted phase contrast microscope	Zeiss	

## Materials and equipment

**Prepare solutions in advance**

• **Phosphate buffered saline (PBS), 1**×: 9.55 g/L in H_2_O (pH = 7.4).

***Note:*** Store at 4°C for up to 2 months.

• **Dextran 5%**: 50 g dextran from *Leuconostoc* spp. in 1 L PBS.

***Note:*** Store at 4°C for up to 2 months.

• **PBS**^**+/+**^: 900 mL PBS + 100 mL Dulbecco’s Phosphate Buffered Saline with calcium chloride and magnesium chloride 10×.

***Note:*** Store at 4°C for up to 2 months.

• **0.4 M CaCl**_**2**_**solution**: 444 mg CaCl_2_ in 10 mL H_2_O.

***Note:*** Store at 4°C for up to 2 months.

• **PBS**^**+/−**^: 50 mL PBS + 125 μL 0.4 M CaCl_2_ solution (final concentration 1 mM CaCl_2_).

***Note:*** Store at 4°C for up to 2 months.

• **PBS EDTA**: 1.46 g EDTA in 1 L PBS (final concentration 5 mM EDTA).

***Note:*** Store at 4°C for up to 2 months.

• PBA-E:

**Table:** 

Reagent	Amount	Final conc.
Bovine serum albumin	250 mg	0.5%
Sodium azide	50 mg	0.1%
PBS EDTA	50 mL	

*Note*: Store at 4°C for up to 1 week.

• RPMI macrophage medium:

**Table:** 

Reagent	Stock conc.	Amount	Final conc.
Heat-inactivated fetal calf serum (FCS)	-	50 mL	10% (v/v)
L-Glutamine	200 mM	5 mL	2 mM
Penicillin-streptomycin	10.000 U/mL and 10 mg/mL	5 mL	100 U/mL and 100 μg/mL
RPMI 1640	-	Fill up to 500 mL	

*Note*: Store at 4°C for up to 4 weeks.

• M1-polarization medium mix, 3×:

**Table:** 

Reagent	Stock conc.	Amount	Final conc.
IFN-γ	20 μg/mL in H_2_O	3 μL	60 ng/mL
LPS	10 μg/mL in PBS	30 μL	300 ng/mL
RPMI macrophage medium	-	Fill up to 1 mL	

*Note*: Prepare fresh each time.

• M2-polarization medium mix, 3×:

**Table:** 

Reagent	Stock conc.	Amount	Final conc.
IL-4	20 μg/mL in H_2_O	3 μL	60 ng/mL
RPMI macrophage medium	-	1 mL	

*Note*: Prepare fresh each time.

• **TPCK-trypsin**: 50 mg trypsin (TPCK-treated) in 50 mL 1 mM acetic acid (final concentration 1 mg/mL).

***Note:*** Store at −20°C for up to 2 years.

• **0.213 M MgCl**_**2**_**solution**: 203 mg MgCl_2_ hexahydrate in 10 mL H_2_O.

***Note:*** Store at 4°C for up to 2 months.

• **EMEM**_**INF**_:

**Table:** 

Reagent	Stock conc.	Amount	Final conc.
MgCl_2_ solution	0.213 M	470 μL	1 mM
CaCl_2_ solution	0.4 M	225 μL	0.9 mM
Albumin solution (BA)	30%	667 μL	0.2%
TPCK-trypsin	1 mg/mL	25 μL	0.25 μg/mL
Optional: Penicillin-streptomycin	10.000 U/mL and 10 mg/mL	1 mL	100 U/mL and 100 μg/mL
EMEM	-	Fill up to 100 mL	

*Note*: Store at 4°C for up to 3 months; add TPCK-trypsin fresh; if penicillin-streptomycin is added, add fresh.

• **Panserin**_**INF**_: 25 μL TPCK-trypsin in 100 mL Panserin 401.

***Note:*** Prepare fresh each time

• **PBS**_**INF**_:

**Table:** 

Reagent	Stock conc.	Amount	Final conc.
MgCl_2_ solution	0.213 M	470 μL	1 mM
CaCl_2_ solution	0.4 M	225 μL	0.9 mM
Albumin solution (BA)	30%	667 μL	0.2%
PBS	-	Fill up to 100 mL	

*Note*: Prepare fresh each time.

• **Plaque medium**:

**Table:** 

Reagent	Stock conc.	Amount	Final conc.
DEAE-Dextran	-	10 mg	0.01%
Albumin solution (BA)	30%	667 μL	0.2%
Sodium bicarbonate	7.5%	2.66 mL	0.2%
Penicillin-streptomycin	10.000 U/mL and 10 mg/mL	1 mL	100 U/mL and 100 μg/mL
MEM 10×	-	10 mL	-
Oxoid agar	3% (in H_2_O)	30 mL	0.9%
TPCK-trypsin	1 mg/mL	25 μL	0.25 μg/mL
H_2_O		Fill up to 100 mL	

*Note*: Prepare fresh each time.

• **PBS**_**Neutral red**_**:** spatula tip of neutral red in 10 mL PBS.

***Note:*** Prepare fresh each time

• **Deuterium-labeled standard mix**:

**Table:** 

Deuterated standard	Molecular weight	Stock conc.	Volume in 50 mL methanol/H_2_O (1:1)	Final conc.
d4-PGE_2_	356.5 g/mol	0.5 μg/μL (1.4 mM)	7.1 μL	200 nM
d5-RvD2	381.5 g/mol	0.1 μg/μL (262 μM)	38.2 μL	200 nM
d8-5(S)-HETE	328.5 g/mol	0.1 μg/μL (304 μM)	32.9 μL	200 nM
d4-LTB_4_	340.5 g/mol	0.1 μg/μL (293 μM)	34.1 μL	200 nM
d5-LXA_4_	357.5 g/mol	0.1 μg/μL (279 μM)	35.8 μL	200 nM
d8-Arachidonic acid	312.4 g/mol	10 μg/μL (32 mM)	15.6 μL	10 μM

*Note*: Store at – 80°C for up to 12 months.

• **TRIS buffered saline**:

**Table:** 

Reagent	Amount	Final conc.
TRIS	151.5 g	6.06%
NaCl	146 g	5.84%
H_2_O	Fill up to 2.5 L	

*Note*: Store at 20°C for up to 2 months.

• **Macrophage lysis buffer**:

**Table:** 

Reagent	Stock conc.	Amount	Final conc.
NP-40	-	1 mL	1%
Na_3_VO_4_	200 mM stock (in H_2_O)	0.5 mL	1 mM
NaF	500 mM NaF stock (in H_2_O)	2 mL	10 mM
Na_4_P_2_O_7_ × 10 H_2_O	-	223 mg	5 mM
ß-glycerophosphate disodium salt hydrate	-	540 mg	25 mM
EDTA	100 mM stock (in H_2_O)	5 mL	5 mM
TRIS buffered saline	-	Fill up to 100 mL	

*Note*: Store at −20°C for up to 12 months.

## Step-by-step method details

### Isolation of monocytes and differentiation and polarization toward M1- and M2 monocyte-derived macrophages (MDMs)

**Timing: >1.5 h for one blood donor (>2.5 h for six blood donors) (for step 1)**

**Timing: >1 h for one blood donor (>1.5 h for six blood donors) (for step 2)**

**Timing: working time >2 h for one blood donor (>3 h for six blood donors); incubation/differentiation time 6 days (for step 3)**

**Timing: >2 h for one blood donor (>4 h for six blood donors) (for step 4)**

**Figure 1:**
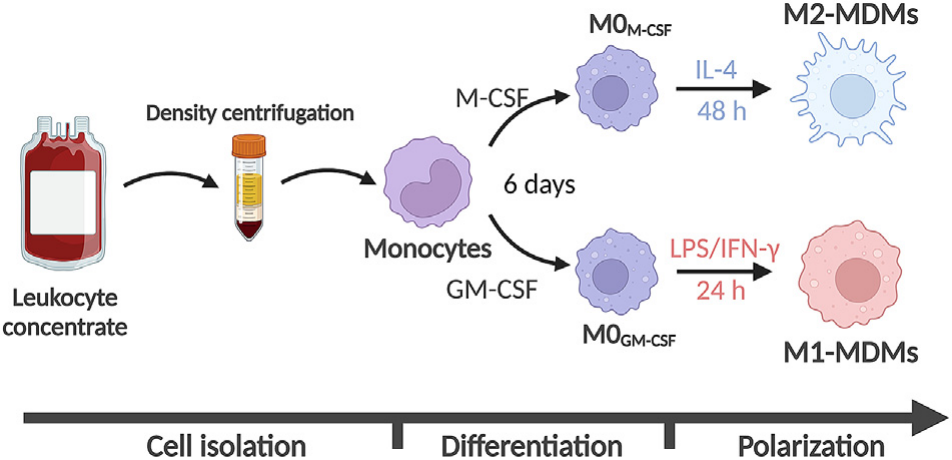
Stepwise protocol scheme Stepwise scheme for 1) monocyte isolation, 2) differentiation to monocyte-derived macrophages (MDMs) and 3) polarization toward M1- and M2-MDMs.

Here, the peripheral blood mononuclear cells (PBMC) are isolated from the leukocyte concentrates via dextran sedimentation and density centrifugation. After counting the cells, they are seeded into cell culture flasks and first differentiated into M0 macrophages and then, after detaching, counting and reseeding them, polarized into either M1- or M2-MDMs. A simplified scheme for the generation of M1- and M2-MDMs from human leukocyte concentrates is shown in Figure 1.

1. Separation of leukocytes by dextran sedimentation and density centrifugation from leukocyte concentrates
  a. Prepare two 50 mL Falcon tubes each containing 10 mL of dextran solution (5%) and 10 mL sterile phosphate-buffered saline (PBS) for each leukocyte concentrate and fill up Falcon tubes with 20 mL blood for a total volume of 40 mL (Figure 2A).

**Figure 2:**
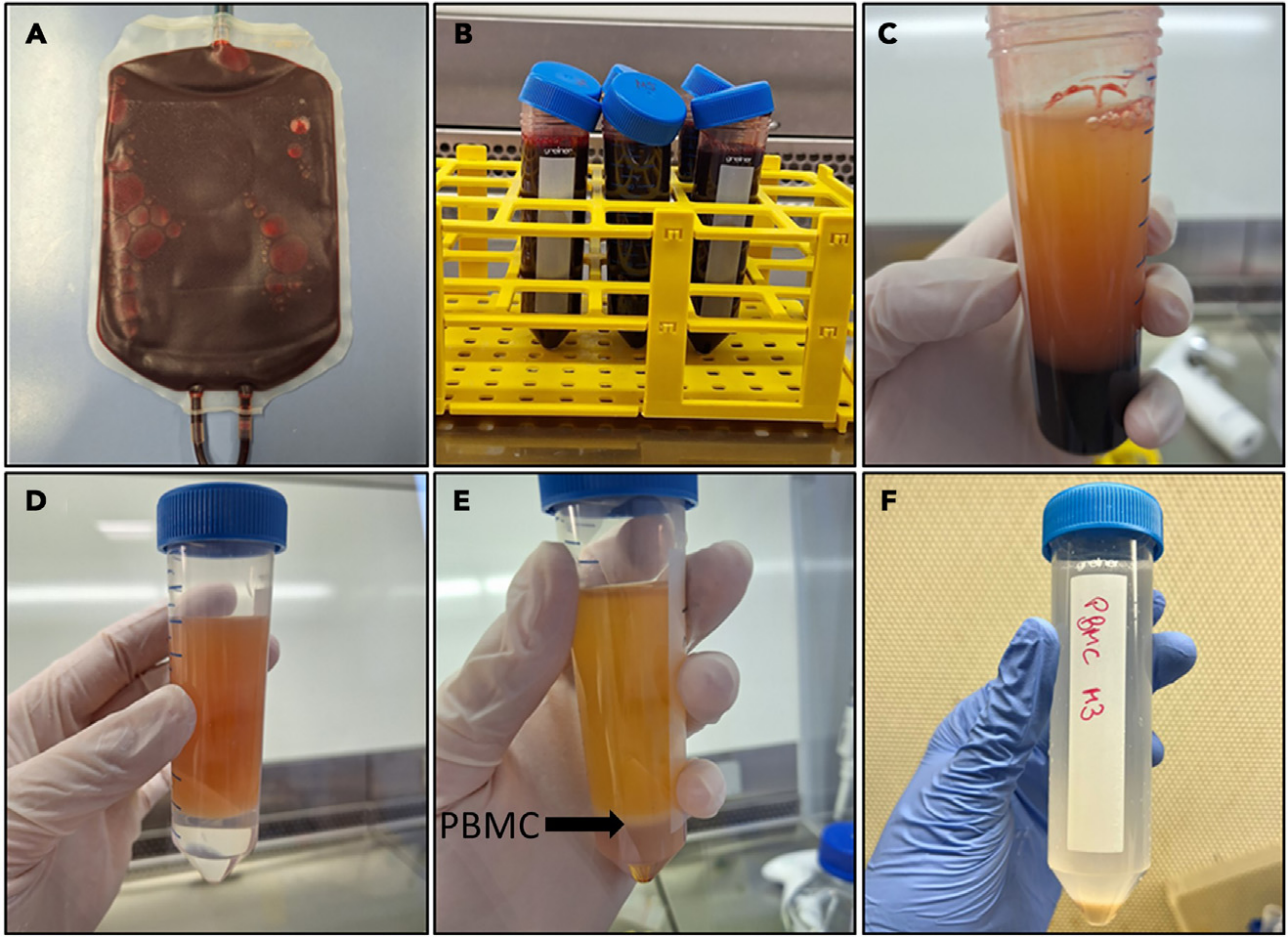
Images of cell isolation process (A) Leukocyte concentrate containing roughly 40 mL of cell-enriched blood. (B) Leukocyte concentrate mixed with dextran/PBS mixture before 40 min of separation. (C) Leukocyte concentrate mixed with dextran/PBS after 40 min of separation. The yellow to orange upper layer contains the white immune cells. (D) Cell rich fraction on top of Histopaque. There is a clear phase boundary between the two layers. (E) Histopaque and cell rich fraction after centrifugation. The white ring, indicated by the arrow, is the PBMC fraction. (F) Pelleted PBMC fraction after centrifugation.
  b. Shake Falcon tubes carefully.
  c. Open the lid and leave to sediment the erythrocytes for 40 min at 20°C (Figure 2B).
  d. Prepare two 50 mL Falcon tubes with 10 mL Histopaque.
  e. Carefully pipet the supernatant from the blood/dextran mixture (color can vary from yellow to orange, Figure 2C) and layer on top of the Histopaque, making sure the two components do not mix (Figure 2D).
    **CRITICAL:** It is easier to avoid mixing the two phases when the pipette controller can be set on lowest speed setting.
  f. Centrifuge at 2,000 rpm (870 × *g*) for 10 min at 20°C without brake (takes around 30 min to stop).
    ***Note:*** We use the Heraeus Multifuge X3R centrifuge.
2. Isolation of the peripheral blood mononuclear cell (PBMC) fraction
  a. The white PBMC fraction (ring) is located between the two liquid phases (Figure 2E). Isolate the cell ring with a pipette from each Falcon tube and combine them in a fresh 50 mL Falcon tube by carefully inserting the pipette tip through the plasma layer.
    **CRITICAL:** It should be avoided to take up the other layers as much as possible. From this step on, keep the cells on ice until you seed them into the flasks.
  b. Fill up to 50 mL with ice-cold PBS and centrifuge at 1,200 rpm (314 × *g*) for 10 min at 4°C (Figure 2F).
  c. Wash the PBMC by first removing/discarding the supernatant, then resuspend the cells in 5 mL ice-cold PBS, and then fill up to 50 mL with ice-cold PBS. Centrifuge at 1,200 rpm (314 × *g*) for 5 min at 4°C.
  d. Repeat step c.
  e. Discard the supernatant and resuspend the pelleted cells in 5 mL ice-cold PBS.
3. Seeding of PBMCs and differentiation of monocytes to macrophages
  a. Count the cells, we observe an average of 4.6 ± 1.5 × 10^8^ PBMC per leukocyte pack (*n* = 48).
    ***Note:*** We use a Vi-Cell XR automated cell counter with a 1:50 cell dilution.
  b. Seed 1.8 × 10^8^ cells into a 175 cm^2^ cell culture flask in 15 mL PBS^+/+^.
  c. Let them adhere for 1 h at 37°C and 5% CO_2_ in the incubator.
    ***Note:*** This will result in the adherence of the monocytes while other cells, especially lymphocytes do not adhere and will be washed away in step d. Morphology of PBMC under the light microscope is shown in Figure 3A.

**Figure 3:**
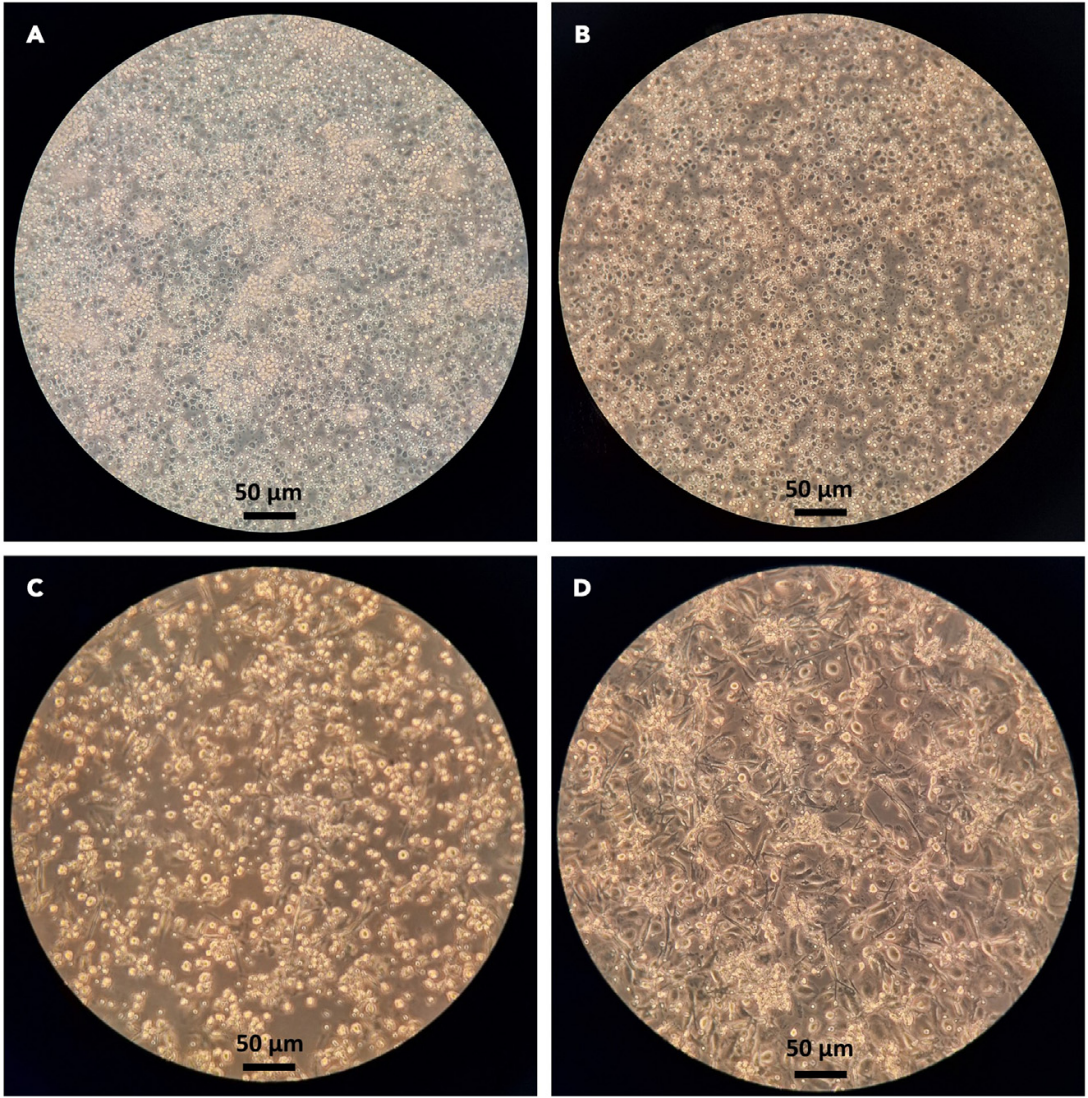
Images of cells under microscope Cells under a 400× microscope (scale bar = 50 μm) for (A) PBMC in PBS^+/+^, (B) PBMC in RPMI macrophage medium, (C) M1-MDMs with round morphology after 24 h polarization and (D) M2-MDMs after 48 h polarization with filamentous morphology.
  d. Remove PBS^+/+^ (containing non-adherent cells) and wash gently with 10 mL pre-warmed (37°C) PBS.
    **CRITICAL:** Never add liquid directly onto the cells, as it will result in cell loss. Rather, pour the liquid onto the side of the flask devoid of any cells.
  e. Add pre-warmed (37°C) 15 mL RPMI macrophage media into the cell culture flask.
  f. Add 60 μL of 5 μg/mL granulocyte/macrophage colony-stimulating factor (GM-CSF stock), final concentration 20 ng/mL GM-CSF, to obtain **M0**_**GM-CSF**_ or 60 μL of 5 μg/mL macrophage colony-stimulating factor (M-CSF stock), final concentration 20 ng/mL M-CSF, to obtain **M0**_**M-CSF**_ to the 15 mL of RPMI macrophage medium.
    ***Note:*** Morphology of PBMC under the light microscope is shown in Figure 3B.
  g. Incubate the cells in the cell culture flask at 37°C and 5% CO_2_ in the incubator.
    **CRITICAL:** For cell culture flasks without air filter in the lid, open the lid slightly.
  h. Remove the medium from the cells and add fresh medium as stated in f) after 3 days.
4. Detachment of M0 macrophages and polarization toward M1- or M2-MDMs
  a. After six days of differentiation, discard the RPMI macrophage medium and gently wash the cells with 10 mL pre-warmed (37°C) PBS.
  b. Add 15 mL pre-warmed (37°C) PBS-EDTA and incubate for 20 min at 37°C in the incubator with 5% CO_2_ to detach the cells from the bottom of the flask.
  c. Gently tap the side of the flasks with your hands to loosen the cells further, then rinse PBS-EDTA over the cells several times to detach most of the cells and collect them into a 50 mL Falcon tube.
  d. Add 10 mL pre-warmed (37°C) PBS to the flask and detach the remaining cells from the bottom of the flask with a cell scraper.
  e. Collect this cell suspension into the 50 mL Falcon tube with PBS-EDTA.
  f. Centrifuge the cells at 1,200 rpm (314 × *g*) for 10 min at 20°C.
  g. Discard the cell supernatant, resuspend the cells in 1 mL RPMI macrophage medium and count the cells.
    ***Note:*** We use a Vi-Cell XR automated cell counter with a 1:10 cell dilution.
  h. Depending on the experimental setup, seed the cells in an appropriate plate (Table 1) in the given RPMI macrophage medium volume, wait for at least 1 h, giving the cells time to attach on the surface.

**Table 1:** Seeding modalities for M0_GM-CSF_ and M0_M-CSF_ according to plate size

Plate	Cell amount	Seeding volume	Polarization mix volume (3×)/well	Total volume/well
6-well	2 × 10^6^	2 mL	1 mL	3 mL
12-well	1 × 10^6^	1 mL	0.5 mL	1.5 mL
24-well	0.5 × 10^6^	0.5 mL	0.25 mL	0.75 mL
96-well	1 × 10^5^	0.1 mL	0.05 mL	0.15 mL
  i. Add the polarization medium for M1- or M2-MDMs, according to Table 1.
    i. M0_GM-CSF_ will be polarized to M1-MDMs for 24 h at 37°C and 5% CO_2_ by adding freshly prepared M1-polarization mix (final concentration: 100 ng/mL lipopolysaccharide (LPS) and 20 ng/mL INF-γ).
    ii. M0_M-CSF_ will be polarized to M2-MDMs for 48 h at 37°C and 5% CO_2_ by adding freshly prepared M2-polarization mix (final concentration: 20 ng/mL IL-4).
  j. For long-term incubations, add the inhibitor or stimulating agent before the M1- or M2 polarization media (usually 30 min).
    **CRITICAL:** Be aware to keep the amount of added DMSO lower than 0.1% of total medium volume.

### Preparations of pathogens

This section will explain how to prepare the pathogens for the stimulation with macrophages. For the live bacteria, this protocol will describe how to generate bacteria in the log-phase and how to prepare bacteria-conditioned medium, which is also a strong agonist for LM biosynthetic enzymes. For the influenza A virus, this protocol, will cover the technique to generate high-concentrated titer stocks for infection of macrophages.

**Timing: >3 days (for step 5)**

**Timing: >3 days (for step 6)**

**Timing: >8 days (including virus stock preparation and determination of the infectious virus particles) (for step 7)**

**Timing: >3 days (for step 8)**

5. Live *Staphylococcus aureus* (*S. aureus*)
  a. Streak *S. aureus* (we use the strain wild type 6850 or wild type USA300) on a Columbia-Agar with sheep blood (5%) plate using a sterile inoculation loop and let the plate grow upside down for 24 h at 37°C.
    ***Note:*** When stored in the fridge at 4°C, the *S. aureus* plate can be used for up to 4 weeks for new liquid cultures.
  b. Pick a single colony from the plate with a sterile pipette tip, put it in 30 mL brain-heart infusion medium (BHI medium) in an Erlenmeyer flask, and let it shake for 24 h at 37°C and 210 rpm.
  c. Measure the optical density (OD) of the culture at 600 nm (use BHI medium as blank).
    ***Note:*** We use an Ultrospec 10 Cell Density Meter from Amersham Biosciences or a BioPhotometer plus from Eppendorf.
  d. Prepare a fresh culture in Erlenmeyer flask with 30 mL BHI medium with an OD of 0.05 and let it shake for 4 h at 37°C and 210 rpm to achieve log-phase.
  e. Centrifuge 15 mL of the liquid culture at 5,300 rpm (3,016 × *g*) for 3 min at 20°C.
  f. Discard supernatant, resuspend the bacteria in 1 mL PBS^+/−^ and dilute until an OD of 1 (PBS^+/−^ as blank) is reached.
6. Live *Escherichia coli* (*E. coli*)
  a. Streak *E. coli* (we use the serotype O6:K2:H1) on a Nutrient Broth (NB)-agar plate using a sterile inoculation loop and let the plate grow upside down for 12 h at 37°C.
  b. Pick a single colony from the plate with a sterile pipette tip, put it in 30 mL NB medium in an Erlenmeyer flask, and let it shake for 24 h at 37°C and 210 rpm.
  c. Measure the optical density (OD) of the culture at 600 nm (use NB medium as blank).
    ***Note:*** We use a Ultrospec 10 Cell Density Meter from Amersham Biosciences.
  d. Prepare a new culture in Erlenmeyer flask with 30 mL NB medium with an OD of 0.05 and let it shake for 3 h at 37°C and 210 rpm to achieve log-phase.
  e. Centrifuge 15 mL of the liquid culture for 3 min at 20°C and 5,300 rpm (3,016 × *g*).
  f. Discard supernatant, resuspend the bacteria in 1 mL PBS^+/−^, and dilute until an OD of 1 is reached.
7. Influenza A virus (IAV)
  a. Propagate and passage the IAV (we use the influenza A virus/Puerto Rico/8/34 (H1N1)) in Madin-Darby canine kidney (MDCK) cells, as described in Karakus et al.^2^
    i. Seed MDCK cells in 15 cm plates one day prior use.
      ***Note:*** MDCK cells should reach 80%–90% confluence 24 h after seeding.
    ii. Remove the medium and wash cells once with PBS. Use a low multiplicity of infection (MOI) (0.01–0.001 MOI, depending on the IAV strain) for initial inoculation in PBS_INF_ for 30 min - 1 h at 37°C and 5% CO_2_ (3–5 mL in a 15 cm plate).
    iii. Remove the inoculum.
    iv. Add a minimal amount of EMEM_INF_ (13–15 mL in a 15 cm plate) for virus propagation and incubate infected cells at 37°C and 5% CO_2_ for 48–52 h, until a cytopathic effect becomes visible.
    v. Sway the plates and/or carefully pipette the medium onto the cell layer to remove sticking virus particles.
    vi. Collect the supernatants and remove cell debris by centrifugation (4,400 × *g*, 10 min, 4°C).
    vii. Store virus aliquots at −80°C and avoid repeated thawing and freezing.
      ***Note:*** If virus replication in EMEM_INF_ medium is inefficient, Panserin_INF_ can be used.
  b. Define the titer of the human IAV strain by standard plaque assay such as described in Karakus et al.^2^
    ***Note:*** To obtain a well-mixed dilution series, vortex each sample gently and use a new pipette tip for each step.
  c. For infection experiments: let the aliquots slowly thaw on ice for 30 min–1 h, depending on the volume of the aliquot.
8. *S. aureus* conditioned medium (SACM)
  a. Follow the same steps as described for 5. live *S. aureus* (step a-e).
  b. Sterile filter the supernatant with a 0.22 μm filter.

**CRITICAL:** Store the SACM at 4°C, never freeze it. SACM can be used for up to 2 weeks.

### Stimulation of M1- and M2-MDMs for lipid mediator profiling

**Timing: 0.5–4 h**

Here, the handling of the MDMs for accurate LM profiling is described. It shows the appropriate stimulation of the cells and how to adequately acquire the samples for LM profiling analysis.

9. Stimulation of macrophages and sample preparation for LM analysis
  a. Assess the differential morphology of the M1- and M2-MDMs under the light microscope:
    M1-MDMs are round and small (Figure 3C), while M2-MDMs are rather oblong (Figure 3D).
  b. Remove polarization medium from the cells.
  c. Immediately add 1 mL PBS^+/−^ when using a 12-well-plate (for stimulation with live bacteria, use volumes according to Table 2).
    ***Note:*** For IAV infection: use PBS_INF_.

**Table 2:** Stimulation of MDMs (displayed for 1 × 10^6^ in 12-well plate) with live bacteria, shown are volume of PBS^+/−^ and volume of bacteria suspension for an MOI of 50

Stimulus	PBS^+/−^ volume	Bacteria suspension	Volume for MOI 50
live *S. aureus*	889 μL	4.5 × 10^8^ bacteria/mL (OD = 1)	111 μL
live *E. coli*	950 μL	1 × 10^9^ bacteria/mL (OD = 1)	50 μL
  d. For inhibitor studies, add the compounds 15–30 min prior to stimulation of the MDMs at 37°C and 5% CO_2_.
    **CRITICAL:** Be aware of keeping the amount of added DMSO lower than 0.1% of total volume.
  e. Stimulate the MDMs for LM production:
    i. For stimulation with live *S. aureus* or *E. coli*, add the stated volume (Table 2) of bacteria suspension to the cells, sway the plate slightly, and incubate the cells for 10–180 min at 37°C and 5% CO_2_.
    ii. For stimulation with IAV, give 5 × 10^6^ IAV to 10^6^ MDMs (MOI of 5) for 30 min in PBS_INF_, then remove the supernatant and incubate the MDMs further for 0.5–4 h at 37°C and 5% CO_2_ in PBS^+/−^.
      ***Note:*** The MOI should be adjusted depending on the study objective and incubation period.
    iii. For stimulation with SACM, add 10 μL of the prepared SACM to the cells, sway the plate slightly, and incubate the cells for 10–180 min at 37°C and 5% CO_2_.
  f. Prepare 2 mL of ice-cold methanol (HPLC-grade) containing 10 μL of deuterium-labeled internal standards for each sample.
  g. Stop the incubation by adding the supernatant of the cells into a new glass vial containing the ice-cold methanol mix.
  h. Vortex the reaction tube and store it at −20°C for at least 2 h to allow protein precipitation before isolation of the lipid mediators.
  i. The subsequent sample preparation and LM extraction by solid-phase extraction and measuring by UPLC-MS/MS is described in Werner et al.^3^

### Phenotyping of M1- and M2-MDMs during host-pathogen interaction

**Timing: >15 min (for step 10)**

**Timing: >15 min (for step 11)**

**Timing: >15 min (for step 12)**

This section shows acquisition of samples for mRNA analysis, protein expression analysis or surface marker determinations. For phenotyping of MDMs, the protein and mRNA levels of LM-biosynthetic enzymes as well as macrophage surface markers in naïve or pathogen-activated MDMs (after step e. in stimulation of M1- and M2-MDMs for lipid mediator profiling) can be exploited.

10. Preparation of macrophage samples for mRNA analysis by qPCR
  a. Remove supernatant from the naïve or activated cells and put the cells on ice.
  b. For RNA isolation, use the E.Z.N.A. Total RNA Kit.
  c. Add 350 μL of the TRK Lysis Buffer (included in the E.Z.N.A. Total RNA Kit) containing 20 μL/mL ß-mercaptoethanol, mix it, and put on ice for 5 min.
  d. Pipet up and down to mix (avoid bubbles), transfer into RNA-free Eppendorf tubes, and freeze at −80°C.
  e. Further preparation for mRNA analysis is described in Jordan et al.^1^ Expected results for mRNA analysis of M1- and M2-MDMs are shown in Figure 4.

**Figure 4:**
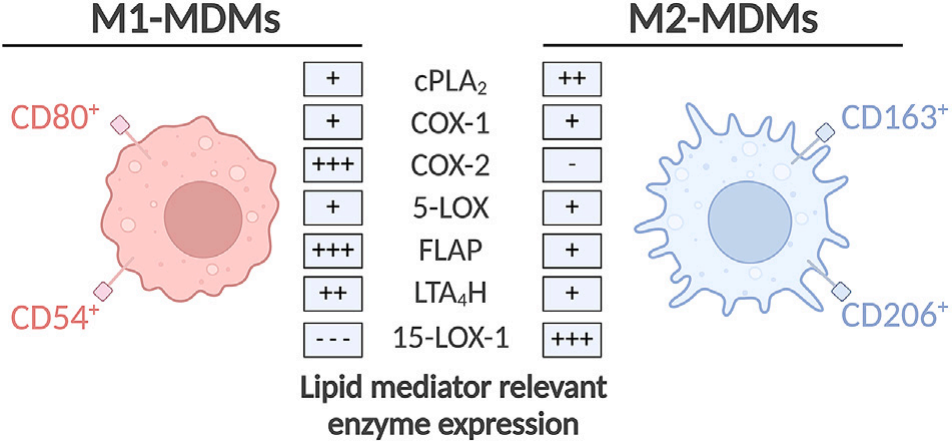
Scheme of expected results for M1- and M2-MDMs Simplified scheme of M1- and M2-MDMs with expected results for specific surface receptors and expression of lipid mediator relevant enzymes according to Peltner et al.^4^ and Werner et al.^3^ The surface markers shown on the M1- and M2-MDMs are characteristic for the respective MDM phenotype. The levels of lipid mediator relevant enzyme expression are characterized as highly expressed (+++), expressed (++), moderately expressed (+), hardly expressed (−) and not expressed (---).
11. Preparation of macrophage samples for LM-biosynthetic enzyme expression on the protein level by Western Blot analysis
  a. Remove supernatant from the naïve or activated cells and put the cells on ice.
  b. Add 100 μL macrophage lysis buffer and keep on ice for 5 min.
  c. Scrape with a pipette tip for at least 1 min, pipet into a 1.5 mL Eppendorf tube and put on ice.
  d. Wait 10 min, vortex, and wait another 10 min, then centrifuge at 4°C, 15,000 rpm (21,130 × *g*) for 10 min.
  e. Transfer the supernatant into a new 1.5 mL Eppendorf tube and freeze at −20°C.
  f. Calculate the protein concentration in the sample.
    ***Note:*** We load 10–20 μg total protein for each SDS-PAGE/western blot sample, depending on the protein of interest by using DC Protein Assay from Bio-Rad.
  g. Further preparation for protein analysis by SDS-PAGE/Western Blot is described in Jordan et al.^1^ Expected results for protein expression of M1- and M2-MDMs are shown in Figure 4.
12. Surface marker determination by flow cytometry
  a. Remove supernatant from the naïve or activated cells and add 1 mL ice-cold PBA-E.
  b. Incubate for 20 min at 37°C to detach the cells.
  c. Detach cells by rinsing gently (avoid bubbles) and pipet into a new Eppendorf tube.
  d. Centrifuge at 4°C, 400 × *g* for 5 min.
  e. Further cell staining for macrophage surface markers by flow cytometry analysis is described in Peltner et al.^4^ Expected expression of surface markers in M1- and M2-MDMs are shown in Figure 4.

## Expected outcomes

Successful PBMC isolation and seeding in PBS^+/+^ should result in cells looking like in Figure 3A. After switching the PBS^+/+^ to RPMI macrophage medium, the cells should look roughly like they do in Figure 3B. When scraping the differentiated cells, it is expectable to gain approx. 3.6 ± 1.3 × 10^7^ cells per donor (*n* = 10) for the M0_GM-CSF_ and 2.3 ± 0.6 × 10^7^ cells per donor (*n* = 10) for the M0_M-CSF_. The yield of the cells can vary, and low cell count is not a definite indicator of poor cell quality. The cells should have a size of 12 μm with a viability of at least 90%. After polarization, the cells should look like those exemplified in Figure 3C for M1-MDMs and in Figure 3D for M2-MDMs. Expected results for M1- and M2-MDMs, regarding surface marker and lipid mediator relevant enzyme expression, are shown in Figure 4.

## Limitations

The presented protocol describes a convenient procedure to attain human monocytes as well as monocyte-derived macrophages. However, we do not claim a pure monocyte cell culture, as the method is not suitable for selective cell-type isolation. Contamination with lymphocytes or platelets in the monocyte fraction is possible. We experienced that not all leukocyte concentrates are suitable for cell isolation. This can be due to, but is not limited to, extreme outside temperatures during transportation above 30°C as well as under 0°C, which can affect cell yield.

## Troubleshooting

### Problem 1

The cells do not separate into a distinct yellow/orange phase (isolation, step 1c (Figure 2C)).

### Potential solution 1

The leukocyte concentrate was stored/transported inappropriately. If this happens, the leukocyte concentrate cannot be used for cell isolation.

### Problem 2

The PBMC fraction does not form a visible white ring between the two layers after density centrifugation (isolation, step 2a).

### Potential solution 2

The cells were pipetted on top of the Histopaque too fast. This will result in distribution of the cells in the bottom layer as there was no clear phase boundary between the Histopaque and the cell suspension before density centrifugation. Make sure to add the cells on top of the Histopaque as slowly as possible without causing turbulences. You can still try to isolate the PBMC by taking the diffuse ring but including a washing step as in isolation, step 2c might be necessary. Alternatively, using Lymphoprep as alternative product compared to Histopaque is possible.

### Problem 3

The isolated PBMC fraction after isolation, step 2b has a rather red color.

### Potential solution 3

The rather red color is a sign of cell impurity, usually the red color indicates erythrocytes being in the same fraction as the PBMCs. Add another washing step as in isolation, step 2c. You can still seed the cells as the erythrocytes are mostly removed with the PBS^+/+^ in isolation, step 3d. Alternatively, also red blood cells lysis buffer can be used.

### Problem 4

The cells do not look like they are shown in Figures 3C and 3D, step 9a.

### Potential solution 4

Screen for macrophage surface markers as shown in Peltner et al.^4^ The specific surface markers for M1-MDMs are CD80 and CD54 and for M2-MDMs CD163 and CD206, as shown in Figure 4. If the cells are positive for the necessary surface markers, the cells can be used. If not, another step of the cell isolation might not have worked well.

### Problem 5

After polarization, there are little to no MDMs at the bottom of the well and there are many floating cells.

### Potential solution 5

• There might be contamination with mycoplasmas, bacteria, or fungi. Discard the cells and decontaminate the lab, a screening with pathogen detection assays is recommended.
• The viability of the cells was too bad already. If there are a lot of dead cells, this can result in a lot more cell death. Discard the cells.

### Problem 6

The stored RPMI macrophage medium has a pink color.

### Potential solution 6

This might point to a possible contamination of the medium and/or oxidized products. Test for microbe contamination, such as mycoplasmas, bacteria, and fungi and check the pH of the medium. If there is no contamination, you can still use the medium.

### Problem 7

The RPMI macrophage medium on the PBMC/MDMs has a yellow color.

### Potential solution 7

This is usually the case when the donor has a lot of cells, leading to a lot of metabolization byproducts that turn the medium’s pH acidic. It’s not a problem as long as the medium is changed as indicated in the protocol. Alternatively, adding larger volume, 30 mL instead of 15 mL, can reduce this issue. However, if most of the cells are dead, this could also indicate a possible contamination.

### Problem 8

During the plaque assay, cell detachment may occur due to cell desiccation or overheated agar medium. This only becomes visible after the staining procedure.

### Potential solution 8

Observe the cells during infection procedure; do not allow the cells to dry out and carefully fill them with medium and agar solution. Also, do not apply too hot plaque medium to the cells.

### Problem 9

Depending on the virus strain and the incubation period, plaques may be very small and difficult to count.

### Potential solution 9

To make plaques clearly visible, we stain the agar with PBS_Neutral red_ for 1 h at 37°C. If the plaques are not clearly visible throughout the agar, agar can be removed using a spatula. If the agar is not solid enough to remove it, incubate the plates for several minutes at 4°C, after that plaques are better visible and have better contrast on the plate. When the agar is removed, the difference/contrast between lysed cells (clear circles) with no background of the red colored agar and living (red colored cells) becomes more visible, making the plaques easier to count. Otherwise, the red-colored agar often shines through and structures are difficult to see.

## Resource availability

### Lead contact

Further information and requests for resources and reagents should be directed to and will be fulfilled by the lead contact, Paul M. Jordan (paul.jordan@uni-jena.de).

### Technical contact

Technical questions should be directed to and will be fulfilled by the technical contact, Paul M. Jordan (paul.jordan@uni-jena.de).

### Materials availability

This study did not generate new unique reagents.

### Data and code availability

This study did not generate any dataset or original code.
